# The American Cancer Society Guidelines on Screening for Breast Cancer: What’s New?

**Published:** 2015-11-01

**Authors:** Pamela Hallquist Viale

After more than 20 years of increased incidences of breast cancer, the year 2000 heralded a welcome statistic: The number of breast cancer cases had begun to decline. The biggest factor in the incidence of breast cancer is thought to be a decline in the use of hormonal therapy after menopause (Chlebowski et al., 2003).

The American Cancer Society (ACS) estimates that in the year 2015, approximately 241,840 new cases of breast cancer will have been diagnosed in women, with 40,290 deaths occurring from the disease. The trend toward a decreased number of cases is good news. Women following the ACS guidelines for early detection of the disease previously received mammograms starting at age 40 and clinical breast exams (CBEs) during provider visits.

## NEW GUIDELINE RELEASED

The recent announcement by the ACS regarding the age at which women should begin mammogram exams has received national attention. The new ACS guidelines state that mammograms should begin at age 45 instead of 40 (although the American College of Obstetricians and Gynecologists recommends age 40 and the US Preventive Services Task Force calls for exams to begin at age 50; Keating &; Pace, 2015), and they call for the elimination of CBEs entirely.

## WHAT DO YOU TELL YOUR PATIENTS WHEN THEY ASK ABOUT SCREENING?

The authors of the new guidelines note that the ACS commissioned a systematic evidence review of the breast cancer screening literature to guide the update, using the quality of this review for the new recommendations (Oeffinger et al., 2015). The evidence demonstrated that screening mammography in women aged 40 to 69 years is associated with a reduced number of breast cancer deaths. However, in younger women, the cumulative lifetime risk of false-positive examinations is greater when mammography is started at a younger age, as the higher recall rate and number of false biopsies in younger women can be a problem. Based on these data, the authors stated that the quality of the available evidence on overdiagnosis is not sufficient to estimate a lifetime risk of breast cancer deaths with confidence.

Therefore, the current ACS recommendations are as follows: Women with an average risk for breast cancer undergo regular screening mammography at age 45, and women from 45 to 54 should receive this screening annually. Women older than 55 should transition to biennial screening or the possibility of continued annual screening. Women from 40 to 44 should have the opportunity to have an annual mammogram screening (presumably after calculating personal risk, family history, and other pertinent factors).

## WHAT ABOUT THE CBE?

The new ACS guidelines do not recommend CBE for breast cancer screening among average-risk women at any age, as the data did not support the practice. Although there are several studies suggesting a possible benefit for the use of CBE in some settings, the extensive review by the authors of the guideline did not definitively show evidence for CBE as an effective screening tool for breast cancer, particularly when mammography is available for patients (Keating &; Pace, 2015; Barton, Harris, &; Fletcher, 1999).

This recommendation may be received with trepidation by some patients who have become accustomed to a CBE within the context of their provider visit. These patients may continue to ask for a CBE, and it’s my personal belief that patients who request the exam should receive one.

As a nurse practitioner, I often use the exam to reinforce techniques of breast self-exam so that patients can replicate the process at home. I also feel that a patient interview can be conducted during a breast exam, using the time to continue to get valuable information regarding the patient’s history while performing a thorough CBE (which can take 5 to 6 minutes; Barton, Harris, &; Fletcher, 1999). The lack of recommendation for CBE in women of any age is certainly a significant departure from previous guidelines—one that will probably engender a number of patient/provider discussions.

## CONCLUSION

Advanced practitioners should incorporate the new guideline as appropriate into their clinical settings. Whether or not there will be insurance coverage for exams conducted outside of the guideline remains to be seen. The ACS guideline does recommend that all women become familiar with the potential benefits, limitations, and harms associated with screening for breast cancer. It is also important to note that women should have the opportunity to begin annual screening between the ages of 40 and 44, as the recommendation to begin screening at age 45 is for women with an average risk of breast cancer.

There is no doubt that screening mammograms have been effective in reducing the number of breast cancer deaths. The good news regarding the incidence of breast cancer is still relevant: The numbers have declined. But the disease remains the second-leading cause of cancer death in women. We must stay vigilant.
